# Deaths of Physicians

**Published:** 1883-10-20

**Authors:** Eugene Foster

**Affiliations:** Chairman Committee on Necrology; Augusta, Ga.


### DEATHS OF PHYSICIANS.

We will publish BRIEF notices of the deaths of medical men. We invite attention to the following note from Dr. Foster, Chairman of Committee on Necrology in the Medical Association of Georgia:

AUGUSTA, GA., August 23d, 1883.

DEAR DOCTOR:

If any member of our Association has died in your County since our last meeting, will you be so kind as to notify me at your earliest convenience.

Respectfully,

EUGENE FOSTER, M.D.,
Chairman Committee on Necrology.

